# Ambient temperature affects postnatal litter size reduction in golden hamsters

**DOI:** 10.1186/s12983-016-0183-8

**Published:** 2016-11-24

**Authors:** Sarah A. Ohrnberger, Raquel Monclús, Heiko G. Rödel, Teresa G. Valencak

**Affiliations:** 1 Institute of Physiology, Pathophysiology and Biophysics, Veterinary University Vienna, Vienna, Austria; 2 Ecologie Systématique Evolution, University Paris-Sud, CNRS, AgroParisTech, Université Paris-Saclay, F-91400 Orsay, France; 3 Laboratoire d’Ethologie Expérimentale et Comparée E.A. 4443, Université Paris 13, Sorbonne Paris Cité, F-93430 Villetaneuse, France

**Keywords:** Lactation, Litter size, Heat dissipation limitation, Juvenile growth, Pup mortality, Early development, Survival, Ambient temperature, *Mesocricetus auratus*

## Abstract

**Background:**

To better understand how different ambient temperatures during lactation affect survival of young, we studied patterns of losses of pups in golden hamsters (*Mesocricetus auratus*) at different ambient temperatures in the laboratory, mimicking temperature conditions in natural habitats. Golden hamsters produce large litters of more than 10 young but are also known to wean fewer pups at the end of lactation than they give birth to. We wanted to know whether temperature affects litter size reductions and whether the underlying causes of pup loss were related to maternal food (gross energy) intake and reproductive performance, such as litter growth. For that, we exposed lactating females to three different ambient temperatures and investigated associations with losses of offspring between birth and weaning.

**Results:**

Overall, around one third of pups per litter disappeared, obviously consumed by the mother. Such litter size reductions were greatest at 30 °C, in particular during the intermediate postnatal period around peak lactation. Furthermore, litter size reductions were generally higher in larger litters. Maternal gross energy intake was highest at 5 °C suggesting that mothers were not limited by milk production and might have been able to raise a higher number of pups until weaning. This was further supported by the fact that the daily increases in litter mass as well as in the individual pup body masses, a proxy of mother’s lactational performance, were lower at higher ambient temperatures.

**Conclusions:**

We suggest that ambient temperatures around the thermoneutral zone and beyond are preventing golden hamster females from producing milk at sufficient rates. Around two thirds of the pups per litter disappeared at high temperature conditions, and their early growth rates were significantly lower than at lower ambient temperatures. It is possible that these losses are due to an intrinsic physiological limitation (imposed by heat dissipation) compromising maternal energy intake and milk production.

## Background

Survival of pre-weaned pups is largely dependent on maternal behaviour, especially in mammalian taxa such as rodents with relatively undeveloped and altricial young that are fully dependent on their mothers for nutrition and thermoregulation early in life [1]. Under natural conditions as well as in laboratory rodents, single pups or entire litters are frequently lost shortly after birth [2]. It has been discussed that factors such as mother’s age and experience, anthropogenically caused disturbance, parity and litter size at parturition play a role [3]. Although dead pups are often consumed by their mothers this does not necessarily imply that the female actively kills them [1]. While some studies exist that tested effects of prenatal heat exposure on later reproductive performance and pup growth after weaning [4, 5], there is only few research investigating effects of ambient temperature on the energy demands of lactating mothers and how this affects survival of pre-weaned young [6–9], particularly in small altricial mammals producing larger litters [10–12].

Effects of ambient temperatures on the survival of pre-weaned, altricial young can act on different levels. Direct influences of extreme temperatures can cause fatal overheating or cooling down of the young, frequently affecting survival of the whole litter [6, 10, 13, 14]. However, temperature-dependent maternal effects can also play a role, through effects on milk production and behaviour of the mothers. It is commonly known that elevated body temperatures and heat stress in livestock animals cause distress and lead to an increased respiration rate, reduced activity and food intake and furthermore to negative effects on breeding performance by reducing fertility [15, 16]. Conceivably, high ambient temperatures have adverse effects on milk production in species such as laboratory mice (*Mus musculus*) [17], common voles (*Microtus arvalis*) [18], Brandt’s voles (*Lasiopodomys brandtii*) [19], Mongolian gerbils (*Meriones unguiculatus*) [20] but also in dairy cattle (*Bos taurus*) [21–24]. Undoubtedly, lactation is the phase when mammalian females show peak metabolic rates, thus having the highest energy consumption and expenditure [25–29]. Recently, various studies have been carried out to identify the “intrinsic” i.e., physiological limits by which females are constrained to further ingest food and transfer nutrients into milk, with consequences for the development of their offspring [17, 30–33]. It becomes clear from these studies that ambient conditions play a key role in this process [6, 18]. We propose that in view of the wide applicability of the concept of heat limiting reproductive outcome, i.e., virtually to all breeding females facing quickly changing thermal conditions and increasingly warm temperatures, physiological mechanisms imposed on mothers may largely affect or even drive individual pup survival and growth. To investigate the associations between such mechanisms and constraints imposed to mothers and their potential impact on offspring survivability, one needs to monitor energy fluxes at different ambient conditions while observing growth and number of all young over the course of lactation and not only at birth and weaning. Under lab conditions, however, such data are often not obtained but rather, focus is given to the number of weaned, viable young, irrespective of the mortality of dependent pups.

When raising larger litters, lactating females undoubtedly reach peak sustained metabolic rates and expend energy at high rates [34–36]. Their body temperature increases [37], their organs work at peak rates, their gastrointestinal tract is widely extended to process and digest even more food [38–40]. Not surprisingly then, females reach a physiological limit where food intake stagnates and cannot be extended any further. Interestingly, the only manipulation that sufficiently enables females to maximise food intake is reducing ambient temperature below the thermoneutral zone i.e., the temperature area where mammals do not need to spend energy on maintenance of their own constant body temperature [30, 41, 42]. At lower ambient temperatures, the females seemingly can raise their energy intake and transform ingested food better into milk as was shown in laboratory mice [41], in striped hamsters (*Cricetulus barabensis*) [43] and in voles [18, 19]. Yet, very often, lactating laboratory mice are exposed to warmer rather than chilled conditions, and it has been argued that laboratory mammals should be housed at 30 °C to best mimic the thermal conditions experienced by humans [44]. However, comparing the thermoregulatory curves of humans and mice suggests that the optimal temperature for single housed mice is in the range of 23 to 25 °C, and around 20 to 22 °C for group housed mice [45].

The general aim of this study was therefore to investigate the link between ambient temperature and pre-weaning pup survival, as reflected by litter size reductions. We conducted this study in golden hamsters (*Mesocricetus auratus*), which are frequently used as pets and as laboratory animals. Golden hamsters are native of the Aleppinian plateau in Syria where they are exposed to huge fluctuations in ambient temperature of −4 °C to 35 °C, not only between summer and winter, but also diurnally during the summer months [46]. Most impressively, they have the shortest gestation period reported in eutherian mammals, of ca. 16–18 days [47]. They give birth to litters of variable sizes of up to 16 young and are therefore expected to produce large quantities of milk until pups reach a self-sustaining state. Apparently, litter sizes do not differ between laboratory strains and wild-derived golden hamsters [48]. Under laboratory conditions, female golden hamsters have been reported to actively reduce litter size [3]. These events have often been described as behavioural pathology and mostly attributed to anthropogenically caused distress, disruption of maternal behaviour [49, 50] or as a reaction to very large litter sizes [3].

We bred female golden hamsters at their thermoneutral (30 °C) and below their thermoneutral zone (22 and 5 °C). We followed the litters from birth to weaning and recorded the occurrence of litter size losses at different ambient temperature conditions. We hypothesised that females kept at lower temperatures would be better able to get rid of excess metabolic heat produced during lactation, and increase their food (gross energy) intake. Therefore, we (*i*) expected to find a positive effect of low ambient temperature conditions on the increase in total litter mass as well as on individual pup growth as a proxy of mother’s lactational performance. However, low temperatures might also give rise to higher associated thermoregulatory costs for mothers and in particular for the offspring, as may be apparent in lower offspring growth [6, 51]. Moreover, we predicted that at lower ambient temperatures (*ii*) females would be able to increase their food intake to cover the increasing energetic demands of lactation. If females can compensate for the higher energetic demands of lactation at lower temperatures (*iii*) they might be better able to raise larger litters, and thus, litter size reductions might be minimised.

## Results

### Litter size reduction

On average, around 37.7% of pups per litter disappeared during the time of study, and such litter size reductions varied between 0 and 100%.

**Fig. 1:**
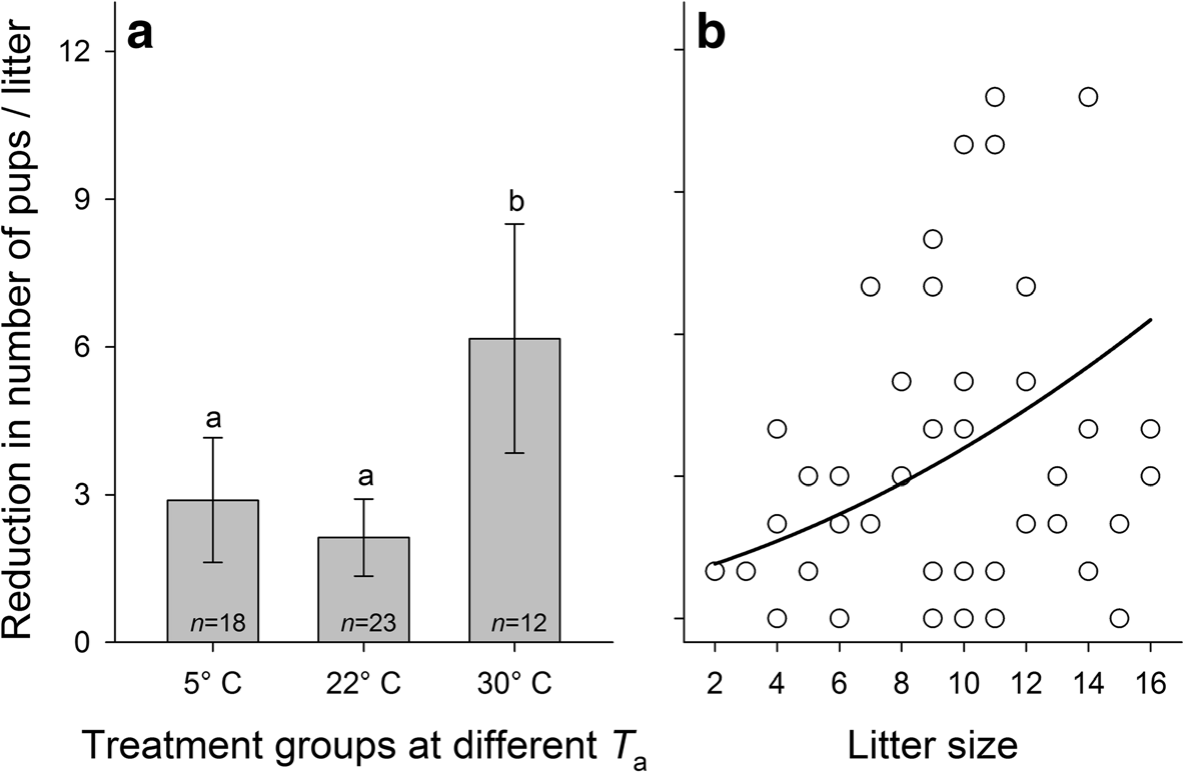
Effects of (**a**) ambient temperature *T*_a_ and (**b**) litter size on litter size reduction. Reduction in the number of pups per litter in (**a**) are given as means ± 95% confidence intervals., Significant pairwise comparisons between ambient temperature conditions (Bonferroni corrected post-hoc comparisons by GLMM for Poisson distributed data) are indicated by different letters. **b** Litter size was quantified on postnatal day 1. Regression line is based on parameter estimates of a multifactorial GLMM for Poisson distributed data; see text for details on statistics

The reduction in the number of pups per litter differed significantly among the mothers kept at the three ambient temperatures (GLMM for Poisson distributed data: *χ*_1_^2^ = 33.10, *P* < 0.001). Litter size reductions were significantly higher when mothers and pups were kept at 30 °C than at 5 °C or 22 °C (post hoc comparisons in Fig. 1a). Furthermore, the reduction in the number of pups was positively associated with litter size, i.e., there was a higher loss of pups in larger litters (*χ*_1_^2^ = 13.94, *β* = 0.126 ± 0.031 SE, *P* < 0.001; Fig. 1b). There was no significant interaction between litter size and ambient temperature on the reduction in the number of pups (*χ*_1_^2^ = 1.90, *P* = 0.39), indicating that the effects of these two predictors were independent from each other.

In addition, a more fine-scaled analysis revealed that the significantly higher litter size reduction in the 30 °C group compared to the 5 °C (*χ*_1_^2^ = 72.48, *P* < 0.001) and 22 °C (*χ*_1_^2^ = 49.80, *P* < 0.001) particularly occurred during the middle part of the lactation period (postpartum days 7–12). Litter size reductions during the early period (postpartum days 1–6) and later period (days 13–19) did not significantly differ between the three ambient temperature conditions (all *P* > 0.10).

There were no significant effects of mother’s body mass and age in any of these analyses (all *P* > 0.10).

### Maternal gross energy intake

We quantified mothers’ gross energy intake (GEI) until postpartum day 10, based on the intake of pellets and mothers’ additional consumption of dead pups. We chose this time interval, as golden hamster pups usually start feeding on solid food at around postnatal day 11, thus making it difficult to quantify the amount of food ingested by the mother within a cage as soon as pups reach this age. Mean values of GEI, averaged over this time span differed significantly among the three ambient temperature conditions (LMM; *F*_2,345_ = 159.94, *P* < 0.001). Pairwise comparisons (all *P* < 0.001) revealed that the GEI including the consumed pups was significantly higher at 5 °C (651.4 kJ/day ± 67.4 SD), intermediate at 22 °C (503.6 kJ/day ± 65.1 SD) and lower at 30 °C (290.1 kJ/day ± 64.8 SD).

**Fig. 2:**
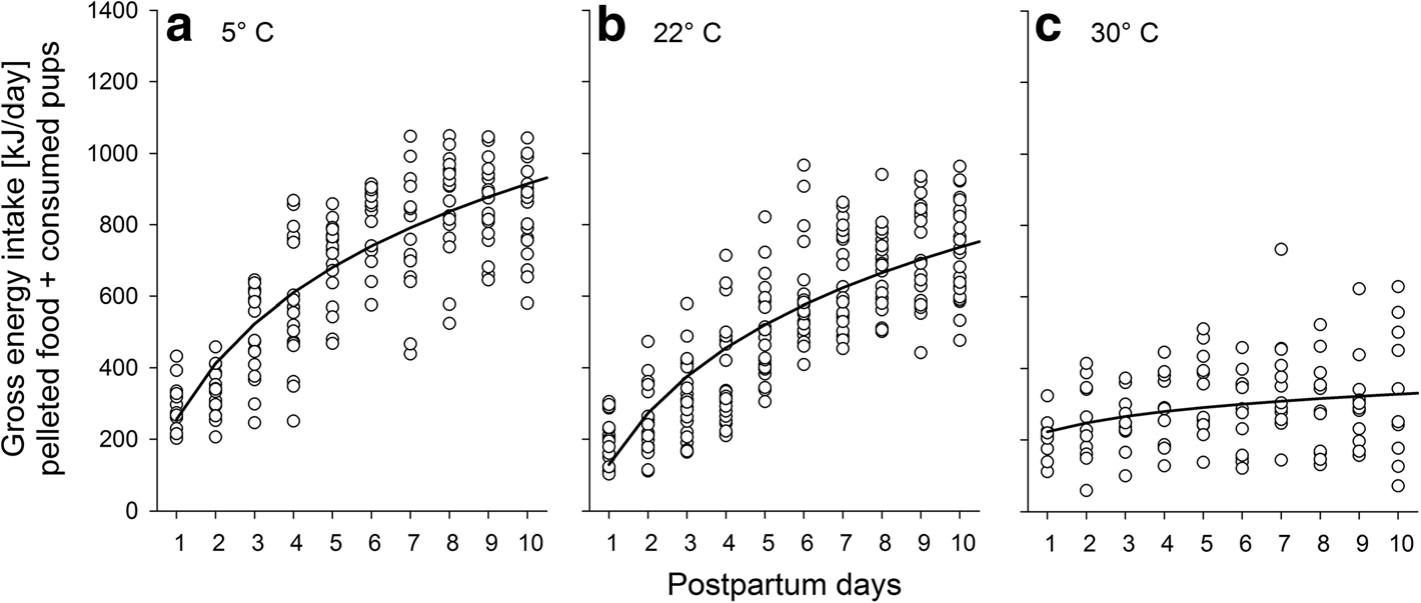
Mothers’ gross energy intake (GEI) during early lactation under different ambient temperature conditions (﻿**a**﻿: 5 °C; **b**: 22° C; **c**: 30 °C). The daily GEI considering the amount of pelleted food and the pups consumed by the mother are given for the first 10 days postpartum, before the young started to feed on pelleted food. Regression lines are based on the back-transformed parameter estimates calculated by LMM; see text for details on statistics

Mothers’ GEI showed differential dynamics over the first 10 days of lactation at the three different ambient temperature conditions (day of lactation × ambient temperature; *F*_2,479_ = 67.61, *P* < 0.001). The energy intake increased significantly during all temperature conditions (5 °C: *β* = 386.73 ± 17.20, *P* < 0.001, Fig. 2a; 22 °C: *β* = 356.53 ± 17.18 SE, *P* < 0.001, Fig. 2b; 30 °C: *β* = 62.44 ± 20.97 SE, *P* = 0.004, Fig. 2c). However, post hoc comparisons revealed that the slopes of these increases were significantly steeper at 5 °C and 22 °C than at 30 °C (all *P* < 0.001), whilst the slopes at 5 °C and 22 °C did not significantly differ from each other (*P* > 0.05). In addition, GEI was significantly and positively associated with the current number of pups alive during the different postnatal days (*F*_1,65_ = 5.69, *β* = 6.18 ± 2.59 SE, *P* = 0.020).

Mother’s body mass and age, or litter size were not significantly associated with GEI in any of these analyses (*P* > 0.10).

### Increases in individual pup mass and in total litter mass

**Fig. 3:**
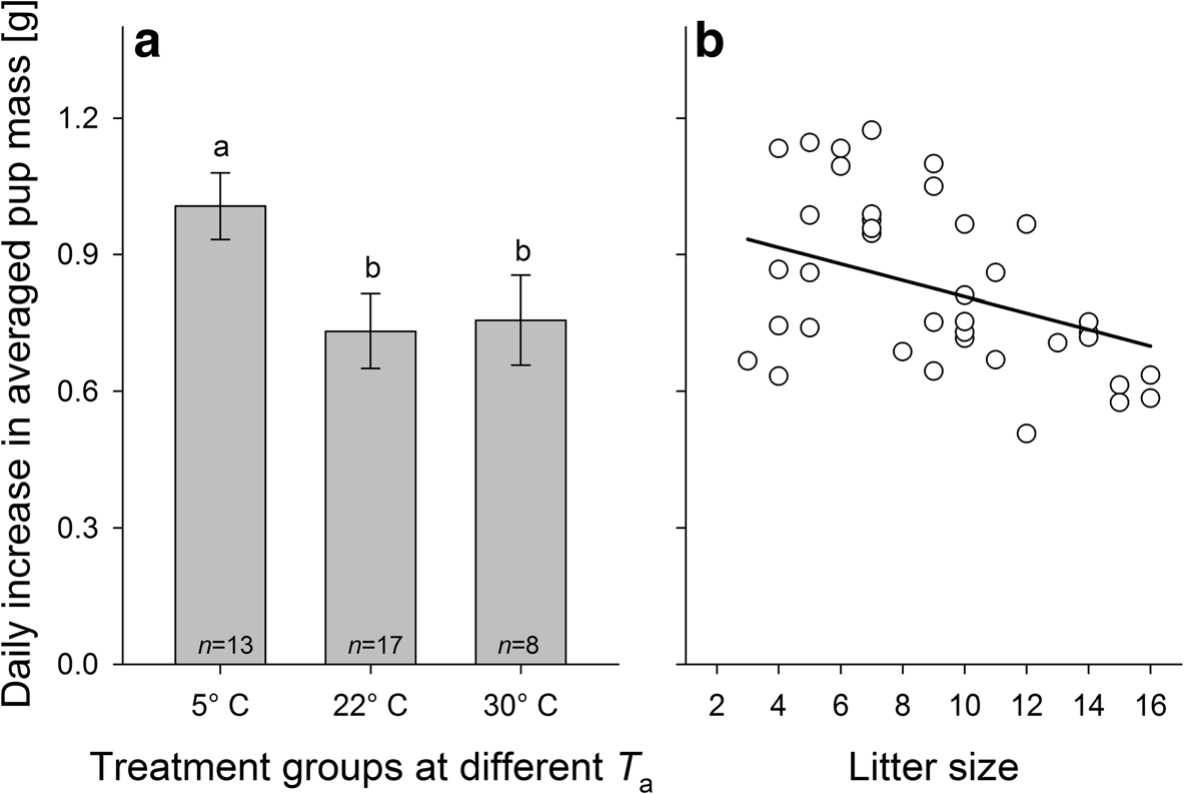
Effects of (**a**) ambient temperature *T*_a_ and (**b**) litter size on the daily increase in average pup body mass. Increases in the averaged individual pup body mass in (**a**) are given as means ± 95% confidence intervals. Significant pairwise comparisons between ambient temperature conditions (Bonferroni corrected post-hoc comparisons by LMM) are indicated by different letters. **b** Litter size was quantified on postnatal day 1. Regression line is based on parameter estimates of a multifactorial LMM; see text for details on statistics. Averaged daily changes in litter mass and in pup body mass were measured between postnatal days 3 and 5; only cases where no pup loss occurred during this time were considered

The daily increase in the individual (averaged) pup body masses, measured between postnatal days 3 and 5, differed significantly between the different ambient temperature conditions (LMM: *F*_2,27_ = 14.56; *P* < 0.001), with significantly higher increases at 5 °C in comparison to the increases observed under 22 °C and 30 °C (post-hoc comparisons in Fig. 3a). Furthermore, the increase in individual (averaged) pup body masses decreased significantly with increasing litter size *F*_1,31_ = 7.87; *β* = −0.018 ± 0.006 SE, *P* = 0.009; Fig. 3b).

Similar results were obtained when total litter masses were analysed. These also significantly differed among the different ambient temperature conditions (*F*_2,33_ = 4.73; *P* = 0.016). Post hoc comparisons revealed significantly higher litter masses at 5 °C in comparison to the litter masses found at 30 °C (*F*_2,33_ = 9.50; *P* = 0.008). There were, however, no significant differences between the litter masses at 5 °C and 22 °C and between the litter masses at 22 °C and 30 °C (both *P* > 0.05). Also here, there was a significant, although positive association between litter mass and litter size (*F*_1,34_ = 58.74; *β* = 0.489 ± 0.064 SE, *P* < 0.001).

There were no significant effects of mother’s body mass and age on the daily increase in litter mass or on the daily increase in the individual (averaged) pup body mass (all *P* > 0.10).

## Discussion

We observed that litter size reduction in golden hamsters from birth to weaning was ambient-temperature dependent, being highest at 30 °C. Furthermore, mothers increased their GEI with decreasing ambient temperature. Moreover, daily increase in litter mass and in pup body mass were higher, the lower the ambient temperature was.

### Litter size reduction

The averaged litter size reduction in our study was 37.7% pups per litter, although with a high variability ranging from 0–100%. Multifactorial modelling revealed that this reduction was partly dependent on initial litter size, with higher reductions occurring in larger litters. Such a higher probability of litter size reduction in larger litters is already well known [10, 52, 53]. This could be mainly attributed to the fact that mammalian mothers of polytocous species typically cannot fully compensate for the increased need of milk production when giving birth to larger litters, thus leading to a lower share of milk available to each individual offspring [52, 54, 55]. Such a negative correlation between litter size and individual pup growth was also confirmed by the results of our study. Furthermore, and as it is has also been suggested for other species of mammals and birds, the probability that mothers adaptively and actively reduce the number of offspring by partial infanticide is increased when litter size is high and offspring’s needs exceed mothers’ available resources [52, 56].

In addition to the observed litter size effects, litter size reductions were highest at an ambient temperature of 30 °C, as exemplified by an average loss of 63.5% of the pups per litter under such conditions. In contrast to our findings, it has been reported for common voles that pup survival was increased at 30 °C, despite experiencing lower growth rates, most likely because pups benefited from the lower maintenance costs at higher ambient temperatures [18]. These contradictory findings in different species of rodents might indicate that at 30 °C, the observation of maternal investment (i.e., negative effects of heat on milk production) and the consequences on the offspring (less energy expenditure for thermoregulation) might be species-specific.

One potential explanation for the observed litter size reduction in the group of the 30 °C mothers could be that heat load or even heat stress might have caused overheating leading to abnormal maternal behaviour or less viability of the offspring, thus resulting in a lowered litter size [13]. Moreover, at 30 °C, reductions were highest around day 7–10 of lactation, that is, at a time point close to peak lactation, when young still rely solely on milk but have a substantially higher demand than at the beginning of lactation [57]. We hypothesize that when peak lactation gets closer and energy demands become even higher, females rather terminate lactation (as manifest in the reduction of pups), than risking hyperthermia.

Litter size reductions might have arisen from several scenarios, which we could not disentangle in this study: Mothers might have actively reduced their litter by killing and consuming their pups [58]. And/or pups might have died and mothers (or even siblings, at least at an older age) might have consumed them. Independently of the imminent causes of death of the pups in the litter, we propose that the reduction in litter size may originate from physiologically imposed energetic constraints that prevent mothers from producing milk at required rates and that reductions in the number of pups were related to metabolism and growth of young cf [57].

### Differences in maternal gross energy intake

Indeed, ambient temperature had a significant effect on mother’s gross energy intake (GEI), which increased over the course of the early lactation period particularly at lower ambient temperatures. In contrast, mother’s GEI remained rather constant, showing a significantly lower increase when animals were housed at 30 °C. One possible explanation might be that mothers exposed to such ambient temperature conditions had notably lower energetic demands than mothers under other temperature treatments.

Maternal GEI in our study also depended on the current litter size through the course of lactation; i.e., females raising larger litters had higher GEIs, and adjusted their GEIs to their changing needs, as it is typically found in small altricial mammals with an income breeding strategy [29, 33, 57]. Further evidence for the income strategy in golden hamsters comes from the fact that maternal body mass, as a proxy of maternal body condition and fat reserves, was not found to be significantly associated to mothers’ GEI in our study. However, such effects have been reported in studies on other species [29, 59], and we cannot exclude that maternal reserves might play a role in this species when food resources are limited.

### Pup growth and increases in total litter mass

In those cases where no litter reduction occurred, pup growth measured during the early postnatal period (days 3–5) was highest at 5 °C. The same result was also apparent with respect to the increase in total litter mass, which could be used as a proxy of mothers’ lactational performance. Pups raised at lower ambient temperatures of 5 °C, just as in our experiment, might have higher energy and maintenance costs for processes such as thermoregulation and growth, which could result in lower growth capacity in those pups leading to lower weaning masses [60]. However, in our study lower temperatures boosted pup growth rates. This is concurrent with other reports showing that mammalian females produce more milk when they are exposed to lower temperatures [18–20, 38, 41, 61, 62]. The rationale behind this effect is that females might face an endogenous physiological limitation imposed by their capacity to dissipate the excess heat produced by the metabolism when organ systems work at peak rates, e.g., during lactation, the undoubtedly most demanding phase in the life of a female mammal [25, 30, 63]. When females are released from this limit, their capacity to metabolise nutrients from food increases [31]. Specifically, these findings suggest that mothers were able to ingest more energy, as in our study, and as a consequence, produced more milk when being released from excessive metabolic heat, for instance through exposure to moderate cold [28, 30, 38, 64]. Interestingly, as this effect was found in our study between postnatal days 3 and 5, it was widely independent of reductions in litter sizes that mostly occurred later on, between postnatal days 7 to 12.

## Conclusions

Taken together, our results suggest that lactating golden hamsters - when housed at ambient temperatures within or above their thermoneutral zone - are limited in their energy allocation to milk production. The ambient-temperature-dependent litter size reduction, which was highest at 30 °C, was probably due to an intrinsic physiological limitation (imposed by heat dissipation), which might have compromised maternal energy intake and milk production. If we accept heat as eminent limiting factor during lactation, females may choose from two options. Firstly, they may risk overheating but still try to maximise GEI with all concomitant consequences. Alternatively, they may limit maternal investment by active litter size reduction (and thus will gain energy by consuming these pups) but rather tolerate the heat. Additional tactics potentially playing a role under natural conditions, such as mother’s active exposure to colder environmental conditions cf. [46], should be explored in further experiments. In conclusion, our study highlights the necessity to carefully consider this result when setting temperature conditions in controlled housing of laboratory rodents as well as of pet animals in private homes.

## Methods

### Animals and housing

Laboratory golden hamsters were obtained from Charles River Laboratories (Sulzfeld, Germany). Using these animals, we started a breeding stock of golden hamsters in our laboratory. From this F1-colony we used a total of 32 females and 10 males in our study. The animals were between 70 and 330 days old, were regularly paired and were allowed to raise litters consecutively (1–4 litters per individual female). We housed them individually in polycarbonate cages (Eurostandard Type IV, 595 × 380 × 200 mm, Techniplast, Germany). Cages equipped with autoclaved wood shavings (Abedd, Ssniff, Germany) were cleaned once a week, unless it coincided with the day of parturition, when females were not disturbed, and special attention was given to late pregnant and early lactating females. All animals were kept on a L:D 16:8 photoperiod. Before and during the pairing all animals were kept at 22 °C. To ensure that all females became pregnant they were paired with males for 4 days, after which the males were removed. Pregnancy was observed by an increase in body mass over 7 days following the mating. On day 7 after mating all pregnant hamsters were randomly assigned to one of three treatment groups with different temperature settings of 5, 22 and 30 °C, and remained under these different temperature regimes until the end of lactation on postnatal day 19. On postnatal day 19 litters were separated from their mothers and all females returned to 22 °C. To expose hamster females to 5 °C, their cages were put in a refrigerated counter (Zoin, Italy), commonly used as counter for cold products offered in supermarkets. That unit warrants constant temperature regulation throughout 24 h a day, has a limited noise production to which the animals easily habituate and finally is open on one side to allow air to flow freely. To generate the 30 °C environment, the animal room was heated up to 30 °C. To monitor stability of ambient temperatures at both 5 °C and 30 °C, we used temperature loggers (DS1921G-F5, Thermochron iButton, Maxim Integrated, USA) with an accuracy of ± 1 °C that were placed in each female’s cage. With the aid of this method, we took measurements every 3 h throughout lactation revealing constant temperatures at all ambient temperature conditions.

### Data collection

All measurements (GEIs, body masses of mothers and pups) were taken daily between 08:00 h am and 11:00 h am. The day when pups were found was considered as the day of parturition, referred to as day 0 of lactation. To minimize disturbances, all measurements were suspended on the day of parturition. Females had ad libitum access to food and water throughout the experiment at all different ambient temperature conditions. Daily food intake (in g) was continuously monitored except during the mating period. To this end, the animals were provisioned with a fixed amount of pelleted food during lactation. Fresh supply was provided and weighed in the morning if necessary. The remaining pellets from the previous day were weighed and the difference (consumption) was noted. All animals received the same diet during the experiment (commercial hamster diet, Ssniff, Germany). The rooms where the experiments took place were accessible only to 4 people, who were following very strict hygiene protocols.

#### Reproduction

Females gave birth approximately 17 to 21 days after introduction of the males and the average litter size observed was 9.3 pups (±3.6 SD; min: 2 pups, max: 16 pups). Interestingly, females gave birth to different litter sizes in the three temperature treatment groups (GLMM for Poisson distributed data: (*χ*_1_^2^ = 15.94, *P* < 0.001). At 22 °C, mothers gave birth to significantly larger litters of on average 11.3 pups (± 3.5 SD). However, mothers had litters with on average 7.8 pups (± 2.8 SD) at 5 °C and 7.8 pups (± 3.3 SD) at 30 °C, which did not differ statistically (pairwise post-hoc comparisons by GLMM).

#### Litter size reduction

Litter size reduction was monitored daily by comparing the number of pups per litter on each given day with the counts on the previous day. It is very unlikely that litter reduction was due to human disturbance, as the animals were used to daily handling and care by us. The fact that we also observed very large litters with few or no reductions further supports this argumentation.

#### Mother’s body mass and gross energy intake

From day 1 of lactation onwards, we continuously measured mother’s body mass, pup number and total litter mass on a daily basis. Measurements of mother’s weights were then averaged within each reproductive event from postpartum day 1 to 19, revealing an average maternal body mass of 144.2 g ± 13.6 SD (min: 117.1 g; max: 187.7 g).

Daily dry food consumption was measured as the difference between the amount (in g) of pelleted food provided to the mother and the remaining amount found in the cage 24 h later. To this end, the cage floor and the bedding (wood shavings) were checked daily for pieces of uneaten food, which were weighed and carefully put back into the cage to not disturb the females’ food hoarding behaviour. In preliminary studies, we observed that these food items can make up a large proportion of the consumed food both by females and pups (late in lactation). Mother’s gross energy intake (GEI) was then calculated by multiplying the daily intake of pelleted hamster food in g/day by its energy content of 16.5 kJ/g (data provided by Ssniff, Germany). Furthermore, we added the energy content of the pups presumably consumed by the mother. For calculation, we assumed an energy content of golden hamster pups of 2.41 kcal/g, as it had been suggested for juvenile laboratory mice of the same age class [31]. In absence of the possibility to measure the energy content of dead hamster pups and in view of the many similarities in juvenile development in rodents in general, we considered this approach both reasonable and justifiable.

Note that between around postnatal days 11 to 13, juvenile golden hamsters usually start picking up solid food themselves (pers. obs. SAO & TGV). Thus, maternal GEI was compared between the different treatment groups only during the first 10 days postpartum.

#### Increase in litter mass and average individual pup growth

As outlined above, we measured total litter mass and the number of pups present daily to the nearest gram. To weigh the litters and to count the pups without largely disrupting maternal behaviour, we carefully took the female out of her cage, first to weigh her (see above) and then gently put her back right after by providing some extra bedding material (unbleached chemical pulp, Pehazell® Hartmann, Germany). This procedure makes females to rapidly pick up and temporarily store the new bedding material inside their cheek pouches. We made use of this short time interval to quickly but gently take out the pups, weigh and count them and carefully put them back into the nest by covering it with the existing bedding. To assess the daily increase in litter mass and in the average pup mass, we compared the litter masses from one day to the next by dividing them by the number of pups in the nest on that particular day.

### Statistical data analysis and sample sizes

All statistical analyses were performed using R, version 3.2.0 [65]. We applied multifactorial linear mixed effects models, LMMs and generalized linear mixed effects models GLMMs for Poisson distributed data by using the R package lme4 [66]. Models always included mother identity as a random factor, as several litters (up to 4) were born from the same mothers. That is, the model structure accounted for repeated measurements of consecutive litters per female. Repeated reproductive events of individual females were assigned to different temperature conditions in the majority of cases (86%) to reduce the chance that single mothers might bias the results by high numbers of repeated measurements within a particular treatment group. We also included parity (i.e., the continuous count of each mother’s reproductive event) in our models as an additional random factor, as maternal performance might potentially differ between consecutive reproductive events [54, 67, 68]. Furthermore, maternal age and maternal mass were included as covariates, as these might potentially affect mothers’ reproductive performance and offspring growth and mortality, as it has been shown in other small altrical mammals [10, 54]. Both covariates were significantly correlated (*R* ^2^ = 0.287, *β* = 0.088 ± 0.019 SE, *P* < 0.001), as mother’s body mass increased with their age. Due to this collinearity, both covariates were never included simultaneously, but we always calculated separate models where we either tested for the effects of maternal mass or maternal age together with all other factor combinations considered (see below). Neither the effects of maternal body mass nor of maternal age were statistically significant in any of our analyses (all *P* < 0.10). Thus, they were removed from the models before these were re-calculated.

Firstly, we tested for the effects of temperature conditions, litter size and maternal mass on the reduction in the number of pups from birth until weaning by GLMM for Poisson distributed data. For this analysis, the entire data set of *N*_litters_ = 53 stemming from *N*_mothers_ = 32 was used. In addition, we carried out a more specific analysis to identify during which part of the lactation period, purported differences in litter sizes reduction between the three ambient temperature conditions were more pronounced. To this end, we separately run the models for the early (postpartum days 1–6), middle (postpartum days 7–12) and late (postpartum days 13–19) lactation period.

Secondly, we analysed the effects of temperature conditions (factor with 3 levels), litter size and maternal mass (both covariates) on maternal GEI during the first 10 days of lactation with LMMs. For a more specific analysis on the dynamics of mothers’ GEI over the period of lactation, litter identity was used as an additional random factor to account for the repeated measurements across the different postnatal days (see Fig. 2). The covariate “postnatal day” was log-transformed to account for the typical non-linear shape of GEI curves across the period of lactation [57]. This analysis was based on 512 daily measurements stemming from 53 litters from 32 different mothers. We statistically compared the slopes of the different increases over time by pairwise calculations (5 and 22 °C; 5 and 30 °C; 22 and 30 °C) of the interactions between postnatal day and ambient temperature.

Finally, we analysed the effects of temperature conditions, litter size and maternal mass on the increase in litter mass and on the average daily pup growth from day 3 to 5 by LMMs. As all litters where mortality occurred during this period were excluded, the remaining sample size was consequently lower (*N*_litters_ = 38 stemming from *N*_mothers_ = 25).

*P*-values of LMMs were obtained by type-II *F*-tests based on the Satterthwaite approximation, and *P*-values of GLMM were obtained by type-II Wald chi-square tests. Non-significant interaction terms were stepwise reduced from the models before these were re-calculated [69]. We assured that the model residuals of LMM were well adjusted to a normal distribution by visually checking normal probability plots. Furthermore, we verified homogeneity of variances by plotting residuals versus fitted values for both, LMM and GLMM [70]. In addition, we added an individual-level random factor to all GLMMs (Poisson) to account for potential effects of overdispersion [71]. All multifactorial models were checked for multicollinearities by calculating variance inflation factors. These were lower than 2 in all cases, indicating no influential effects of multicollinearities [72]. For all significant covariate effects, the slope (*β*) of the association including its standard error are given as a measure of effects size.

## Abbreviations

T_a_: Ambient temperature
GEI: Gross energy intake
GLMM: Generalized linear mixed effects models
LMM: Linear mixed effects models
T_a_: Ambient temperature
